# Factors associated with the performance and achieving goals of specialized periodontal procedures in Brazilian dental specialties centers

**DOI:** 10.1186/s12913-024-10776-9

**Published:** 2024-03-08

**Authors:** Raíres Chaves da Silva Rodrigues, Yuri Wanderley Cavalcanti, Edson Hilan Gomes de Lucena

**Affiliations:** Federal University of Paraiba, João Pessoa, Brazil

**Keywords:** Secondary health care, Health care assessment, Oral health, Quality indicators in health care

## Abstract

**Background:**

The Brazilian Dental Specialty Centers (CEO, in Portuguese) represent the strategy of the National Oral Health Policy to provide secondary-level dental care. They offer more complex procedures, such as the treatment of periodontitis. This study aims to investigate the factors associated with the performance and the achievement goals of specialized procedures and the achievement gols of periodontics in CEO.

**Methodology:**

Analytical and cross-sectional study using secondary data. The database of the second cycle of the External Evaluation of the National Program for Improving Access and Quality in CEO (PMAQ-CEO, in Portuguese), was utilized, which assessed 1,042 CEO on-site in 2018. The data were analyzed using multiple Poisson regression, estimating the prevalence ratio (PR) (*p* < 0.05).

**Results:**

A third of the CEO (*n* = 305) performed all specialized procedures, with a higher prevalence observed in those with more than one bicarbonate jet prophylaxis unit (RP = 2.12; 95% CI: 1.160–3.881; *p* = 0.015) and when they had a higher percentage of specialist professionals (RP = 1.004; 95% CI: 1.002–1.006; *p* < 0.001). The periodontics goal was achieved by 617 (59.2%) CEO, with a higher prevalence among those who had a manager with supplementary training (PR = 1.21; 95% CI: 1.100-1.335; *p* < 0.001) and with a higher workload for the periodontist dentist (PR = 1.15; 95% CI: 1.103–1.201; *p* < 0,001).

**Conclusion:**

Although most CEOs do not perform allspecialized periodontics procedures, more than half achieved the established goals. The provision of specialized periodontics services in CEO and the achievement of goals are influenced by the quantity and professional qualifications, as well as the availability of equipment.

The need to analyze compliance with periodontics goals in the Brazilian Dental Specialties Centers (CEO, in Portuguese) is justified, as there are no studies in this area with this specific approach, Brazilian Dental Specialties Centers making it an unprecedented analysis. Moreover, it is necessary to consider the critical role of CEO in relation to the attention/control of periodontal disease, considering that, in terms of public policy, it corresponds to the only access for most Brazilians to periodontal care at a specialized level. The aim of this study is to investigate, at a national level, which individual factors are associated with the achievement of goals in the periodontics specialty in Brazilian CEO.

## Introduction

Periodontal diseases represent a significant public health concern, with the potential to have a substantial impact on the well-being of numerous individuals worldwide [1]. This condition is associated with a decreased quality of life and can lead to chewing dysfunctin, resulting in significant consequences for oral health maintenance. Approximately 11% of the global population faces this public health challenge, with its highest prevalence typically occurring approximately 40 years old, a critical age group within the economically active population [2].

The Brazilian Oral Health Survey (Projeto Saúde Bucal Brasil, in Portuguese) is an investigation into the oral health conditions of the Brazilian population. The results of the most recent survey conducted in 2010 reveal that the prevalence of periodontal problems varies by age group. The rate of affected individuals is 37% at 12 years old, 49.1% in the 15 to 19-year-old range, 82.2% among adults aged 35 to 44, and 98.2% in individuals aged 65 to 74. The presence of dental calculus and bleeding is more common among 12-year-olds and teenagers [3].

Brazilian Oral Health Teams (BOHTs) working in primary healthcare have the responsibility to ensure that the population has timely access to the prevention and proper treatment of periodontal disease, and should solve more than 80–85% of the population’s health problems. This is essential for effectively controlling the disease, improving the quality of life, and preventing its progression [4].

The Dental Specialty Centers (CEO, in Portuguese) represent the strategy of the Brazilian Oral Health Policy to provide secondary-level dental care. They offer more complex procedures, such as the treatment of periodontitis. This involves subgingival root planing and scaling, as well as access surgery. Periodontal surgery is also performed for restorative dentistry purposes, including gingivectomy, crown lengthening by flap, and procedures related to increasing gingival volume. These centers also treat furcation lesions [5].

The Ministry of Health (MoH) has established productivity targets for various specialties (endodontics, periodontics, minor oral surgery, and patients with special needs) based on the classification of CEO. Specifically, for Periodontics, the minimum monthly targets are 60, 90, and 150 procedures for types I, II, and III CEO, respectively. Failure to meet these production targets for two consecutive months or three alternating months within a year may result in the suspension of monthly incentives for CEO [6];[7].

However, in addition to offering greater access to secondary care, it was necessary to invest in evaluation processes at this level of care. For this purpose, the MoH implemented the External Evaluation of the Program for Improving Access and Quality (PMAQ-CEO). Through Ordinance GM/MS No. 261, of February 21, 2013[8]. As well as the Self-Assessment for Quality Improvement manual (AMAQ-CEO in Portuguese), which brought the self-assessment forms and worksheets to intervention plans [9].

In this context, assessment plays a crucial role as a management tool that enables the analysis of the effectiveness of secondary care within the healthcare network. Furthermore, it raises questions about access, service functionality, and its relationship with other levels of care [10];[11]. In terms of goal achievement, studies have indicated that the Human Development Index (HDI), professional work hours, equipment availability, referral system, infrastructure, and management may be correlated [12];[13].

This study aims to investigate the factors associated with the performance of specialized procedures and the achievement of goals in periodontology at Brazilian CEO.

## Methods

### Ethical aspects

The External Evaluation of the National Program for Improving Access and Quality in CEO (PMAQ-CEO), *Programa Nacional de Melhoria do Acesso e da Qualidade dos Centros de Especialidades Odontológicas* was approved by the Research Ethics Committee (CEP) of the Federal University of Pernambuco (UFPE), under CAAE number 23458213.0.0000.5208, complying with the requirements of Resolution n.466/12 of the National Health Council.

### Study design

This is an analytical cross-sectional study using secondary data. The data used in this study were extracted from the second cycle of the PMAQ-CEO. This program involved an on-site survey to assess the access and quality conditions of 1,097 CEO distributed throughout the country, whether or not they had participated in the program. However, 55 CEO were excluded from the evaluation due to ongoing renovations, being disabled, or refusing to participate in the external evaluation. Therefore, the present study analysed 1,042 CEO, 95% the inclusion rate, who were accredited by the Brazilian Ministry of Health in 2018.

### Data collection

The microdata used in this study were obtained from National Information Systems with public and unrestricted access.

The universe of the study comprises the municipalities in which the CEO adhered to the PMAQ-CEO and who answered the external evaluation questionnaire. Of the CEO implemented in the Brazilian territory in 2018, 1097 answered the external evaluation questionnaire. Of these, 45 were excluded for showing zero production in all months of the year, 05 for not having identified the type of CEO and 05 for not providing individual data in the database of the PMAQ-CEO 2nd cycle. Therefore, the final sample consisted of 1,042 (95%) CEO from 809 municipalities in Brazil. The authors did not had access to information that could identify individual participants during or after data collection.

A researcher collected data for Modules I - Observation at the CEO and II - Interview with the CEO Manager, CEO Dentist, and Document Verification. The data were made available by the MoH (https://www.gov.br/saude/pt-br/composicao/saps/pmaq/ciclos-do-pmaq-ceo/2-ciclo-ceo/instrumentos-de-avaliacao-externa).

### Variables

**Fig. 1:**
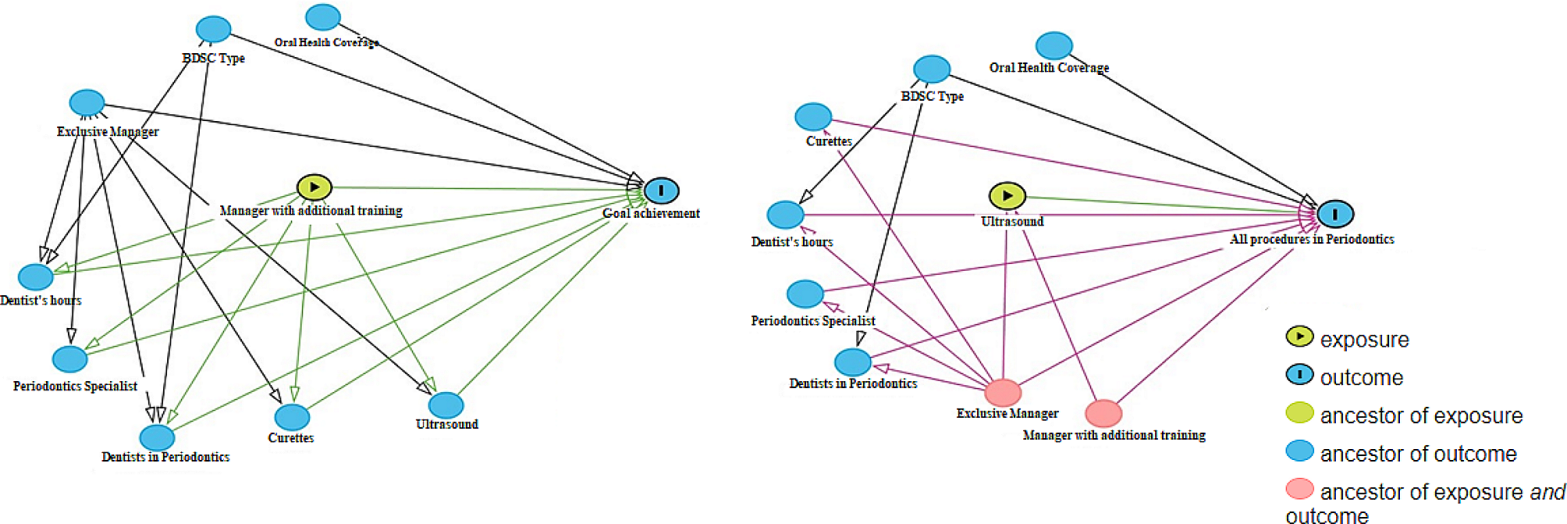
Directed acyclic graphs (DAG) depicting hypotheses about the relationships between exposure, covariates, and outcome (completion of all specialized procedures and achievement of the periodontics goal)

The dependent variables considered in the study were completion of all specialized periodontal procedures (treatment of periodontitis; subgingival scaling and root planing; access surgery; periodontal surgery for restorative dentistry; gingivectomy; clinical crown lengthening by flap; treatment of furcation lesions - grade I, II, and III; procedures for controlling/removing excess gingival tissue, and gingival graft), dichotomized as “yes” or “no”. Achievement of the quantitative target established by the MoH for periodontology, dichotomized as “achieved” or “not achieved” (Fig. 1).

The independent variables used were oral health coverage in the Family Health Strategy in 2018, dichotomized by the median (< 57% and > = 57%); type of CEO (I, II, and III); the number of air-polishing prophylaxis devices in usable condition (none, only one, or more than one); availability of Gracey curettes in usable condition (yes or no); the presence of a manager exclusively dedicated to CEO management (yes or no); additional training of the manager (yes, in public health/community health, no, or no manager); percentage of periodontics professionals with specialization in the field; total weekly workload of the periodontist; and the number of periodontists (Fig. 1).

### Data analysis

Two directed acyclic graphs (DAGs) (Fig. 1) were created using the DAGitty software program (version 3.0) to examine the relationships between dependent and independent variables and to identify potential sources of bias and uncertainty in the results of an epidemiological study [14].

Initially, the data were analyzed using descriptive statistics to characterize the study population, obtaining absolute frequencies (n), relative frequencies (%), means, and standard deviations. An exploratory analysis was conducted using the Pearson chi-squared test to examine associations between variables, assessing the presence or absence of statistically significant differences. Subsequently, a robust Poisson multiple regression model was obtained to determine the prevalence ratio (PR) of the outcomes. Independent variables were individually analyzed in a bivariate manner with the outcomes, and variables with *p* < 0.20 were included in the initial model, with those with *p* < 0.05 remaining in the adjusted model. Confidence intervals of 95% were calculated for the variables under study. The data were tabulated and analyzed using JAMOVI software.

## Results

The performance of all specialized Periodontics procedures was associated with type III CEO (39%; *p* = 0.014), having more than one bicarbonate jet prophylaxis device (37.1%; *p* = 0.001), and CEO who had managers exclusively dedicated to management, at (33.7%; *p* = 0.020). Furthermore, the CEOs who performed all specialized periodontal procedures had a higher average percentage of periodontal professionals with specialization (68.2), sum of the weekly working hours of the periodontist dentist (31.6) and number of dentists practicing in the specialty of periodontics (1.5). (Table 1).

**Table 1:** Descriptive analysis and chi-square test to assess the association between the variables and the completion of all specialized periodontics procedures at the CEO, 2018, Brazil

			The CEO performs all specialized periodontics procedures.						
			Yes			No			
**Variables**			**n**	**%**	**Mean (Standard deviation)**	**n**	**%**	**Mean (Standard deviation)**	**p value**
Oral Health Coverage		< 57%	161	30.8		361	69.2		0.264
		>= 57%	144	27.7		376	72.3		
CEO Type		I	108	25.8		310	74.2		0.014
		II	144	29.5		344	70.5		
		III	53	39.0		83	61.0		
Prophylaxis devices with bicarbonate jet		None	10	15.6		54	84.4		0.001
		One	107	22.7		364	77.3		
		More than one	188	37.1		319	62.9		
Gracey curettes		Yes	297	29.8		700	70.2		0.083
		No	8	17.8		37	82.2		
CEO has a manager who works exclusively in management		Yes	136	33.7		267	66.3		0.020
		No No Manager	160 10	27.1 20.8		431 38	72.9 79.2		
The manager has a background in Public Health/Public Management		No manager	9	18.8		39	81.3		
		Yes	137	34.1		265	65.9		0.015
		No	159	26.9		433	73.1		
Percentage of periodontal professionals with specialization					68.2 ($\pm$43.5)			55.9 ($\pm$47.5)	
Sum of the weekly working hours of the periodontist dentist					31.6 ($\pm$20.7)			31.4 ($\pm$28.2)	
Number of dentists practicing in the specialty of periodontics					1.5 ($\pm$0.9)			1.3 ($\pm$0.8)	

*P* < 0,05

The CEO located in municipalities with lower oral health coverage (62.8%; *p* = 0.017), of type III (69.1%; *p* = 0.016), who had more than one bicarbonate jet prophylaxis device (66.5%; *p* = 0.001), with a manager exclusively focused on management (63.3%; *p* = 0.047), and with a background in Public Health or Public Management (68.2%; *p* = 0.001) achieved more periodontal goals. (Table 2).

**Table 2:** -Descriptive analysis and chi-square test to verify the association between variables and the achievement of periodontics goals in CEO, 2018, Brazil

		Achievement of Periodontics Goals							
			**Achieved**				**Not achieved**		
**Variables**		**n**	**%**	**Mean (Standard deviation)**	**n**	**%**		**Mean (Standard deviation)**	**p value**
Oral Health Coverage	< 57%	328	62.8%		194	37.2%			
	>= 57%	289	55.6%		231	44.4%			0.017
CEO Type	I	231	55.3%		187	44.7%			0.016
	II	292	59.8%		196	40.2%			
	III	94	69.1%		42	30.9%			
Prophylaxis devices with bicarbonate jet	None	32	50.0%		32	50.0%			0.001
	One	248	52.7%		223	47.3%			
	More than one	337	66.5%		170	33.5%			
Gracey curettes	Yes	595	59.7%		402	40.3%			0.150
	No	22	48.9%		23	51.1%			
CEO has a manager who works exclusively in management	Yes	255	63.3%		148	36.7%			0.047
	No	339	57.4%		252	42.6%			
	No manager	23	47.9%		25	52.1%			
The manager has a background in Public Health/Public Management	Yes	274	68.2%		128	31.8%			0.001
	No	320	54.1%		272	45.9%			
Percentage of periodontal professionals with specialization				61.8 ($\pm$45.6)				56.1 ($\pm$48.1)	
Sum of the weekly working hours of the periodontist dentist				33.6 ($\pm$27.3)				28.0 ($\pm$23.7)	
Number of dentists practicing in the specialty of periodontics				1.5 ($\pm$0.9)				1.2 ($\pm$0.7)	

*P* < 0,05

According to the adjusted Poisson regression model (Table 3), the factors related to the performance of all specialized periodontics procedures by CEO were the presence of bicarbonate jet prophylaxis devices and the percentage of periodontics specialists. CEO who had more than one bicarbonate jet prophylaxis device had a higher prevalence (RP = 2.12; 95% CI 1.160–3.881; *p* = 0.015) than those who had only one or no such device. Regarding the percentage of periodontics specialists, a higher percentage was associated with a higher prevalence of performing all specialized procedures (RP = 1.002; 95% CI 1.002–1.006; p = < 0.001) compared to those who were not specialists.

**Table 3:** Poisson multiple regression model of factors related to the performance of all specialized periodontics procedures in the CEO, 2018, Brazil

	Crude Model				Adjusted Model			
Variables	B	p value	PR	95% CI	B	p value	PR	95% CI
Oral Health Coverage - >= 57%	-0.103	0.293	0.902	0.745–1.093			-	
Oral Health Coverage - <57%			1				-	
CEO– **Type III**	0.359	0.008	1.432	1.099–1.866			-	
CEO– **Type II**	0.075	0.493	1.078	0.869–1.337			-	
CEO– **Type I**			1				-	
Bicarbonate jet– **More than one**	0.742	0.016	2.101	1.150–3.838	0.752	0.015	2.122	1.160–3.881
Bicarbonate jet-**One**	0.302	0.335	1.353	0.732–2.502	0.341	0.279	1.406	0.759–2.603
Bicarbonate jet**- None**			1				1	.
Gracey curettes - **No**	-0.392	0.218	0.676	0.362–1.260			-	
Gracey curettes - **Yes**			1				-	
CEO with manager exclusively– **No**	-0.209	0.031	0.811	0.671–0.981			-	
CEO with manager exclusively– **Yes**			1				-	
Manager with Public Health/Public Management– **No**	-0.202	0.037	0.817	0.676–0.988			-	
Manager with Public Health/Public Management– **Yes**			1				-	
Professionals with specialization	0.004	< 0.001	1.004	1.002–1.006	0.004	0.001	1.004	1.002–1.006
Weekly working hours of the periodontist dentist	0.000	0.944	1.000	0.997–1.003			-	
Dentists practicing in the specialty of periodontics	0.148	0.004	1.159	1.049–1.281			-	

*P* < 0,05

Regarding the factors related to the achievement of periodontics goals in CEO, in the adjusted multiple Poisson regression model (Table 4), a significant difference (*p* < 0.05) was observed. Showing a higher prevalence when the manager had a background in Public Health or Public Management (RP = 1.212; 95% CI 1.100-1.335; *p* < 0.001) and when the weekly working hours of the periodontist were higher (RP = 1.151; 95% CI 1.103–1.201; *p* < 0.001).

**Table 4:** Poisson multiple regression model of factors related to the achievement of the periodontics goal in the centers, 2018, Brazil

	Crude Model				Adjusted Model			
Variables	B	p value	PR	95% CI	B	p value	PR	95% CI
Oral Health Coverage- **>=57%**	-0.122	0.017	0.885	0.801–0.978			-	
Oral Health Coverage - **<57%**			1				-	
CEO– **Type III**	0.166	0.021	1.181	1.026–1.359			-	
CEO– **Type II**	0.049	0.388	1.050	0.940–1.174			-	
CEO– **Type I**			1				-	
Bicarbonate jet– **More than one**	0.160	0.198	1.174	0.920–1.498			-	
Bicarbonate jet– **One**	-0.044	0.731	0.957	0.745–1.230			-	
Bicarbonate jet– **None**			1				-	
Gracey curettes– **No**	-0.169	0.280	0.845	0.622–1.147			-	
Gracey curettes– **Yes**							-	
CEO with manager exclusively– **No**	-0.093	0.065	0.911	0.825–1.006			-	
CEO with manager exclusively– **Yes**			1				-	
Manager with Public Health/Public Management - **Yes**	0.232	< 0.001	1.261	1.141–1.393	0.192	< 0.001	1.212	1.100-1.335
Manager with Public Health/Public Management - **No**			1				1	
Professionals with specialization	0.001	0.039	1.001	1.000-1.002			-	
Weekly working hours of the periodontist dentist	0.002	0.007	1.002	1.001–1.004	0.140	< 0.001	1.151	1.103–1.201
Dentists practicing in the specialty of periodontics	0.143	< 0.001	1.154	1.106–1.204			-	

*P* < 0,05

## Discussion

The study demonstrated that the performance of specialized periodontal procedures was associated with the presence of CEO that had bicarbonate jet prophylaxis devices. Previous studies have already shown that the use of ultrasonic instruments, compared to periodontal curettes, reduces treatment time and increases the number of patients treated [15].

New instrumentation methods have been developed to achieve excellent clinical outcomes in a shorter time frame. Manual instrumentation typically requires more time in the office to achieve similar levels of effectiveness compared to ultrasonic instruments [16]. The use of the latter can significantly reduce the needed time, with reductions of up to 50% [17]. These results are consistent with other studies and indicate that, in comparison to manual instruments, ultrasonic instruments can statistically decrease treatment time by over 30% [15].

Another variable with a positive impact on the completion of all specialized procedures by the CEO was the proportion of periodontics specialists. When a dentist has specialization and works in their specific area, the workflow becomes more efficient and agile. This results in an increase in the number of appointments and a reduction in the waiting list. As a consequence, the service can achieve the goals set by the Ministry of Health [18];[19].

It is of great importance for the service to have specialized professionals and to provide longer working hours for those specialists. This should result in a greater variety of specialized periodontal procedures being offered, as well as an increased production of these procedures. Although, as a public policy, it may not directly measure quality, it can impact the population’s access and the profile of specialized services offered to the user [20].

The research conducted by Galvão in 2021[21] revealed that the quality of secondary oral health care was positively influenced by the workload and the number of specialized professionals in each area. According to the authors, these factors explain the differences in the performance of municipalities regarding the provision of specialized dental services.

Regarding the achievement of goals, one of the significant factors in the present study was the presence of a manager dedicated exclusively to the area, along with additional training in Public Health/Public Management. A study conducted by Lucena and colleagues in 2019[22] found that in CEO where managers exclusively performed this role and had additional training in public management or public health, there was a notable increase in the execution of planning activities, resulting in a higher and more efficient number of appointments.

In this context, it becomes crucial for CEO to have managers, preferably dedicated solely to the administration of the service, who possess the necessary skills to carry out planning, self-assessment of the service, and rigorous monitoring of established goals. However, it is essential to emphasize that these activities and actions should not be seen merely as bureaucratic procedures devoid of meaning; they should involve managers and professionals who are directly involved in the provision of services and care [22].

However, it is important to highlight that the situation in Brazil presents significant challenges in practice, especially in public healthcare institutions. The task of effectively training managers faces considerable obstacles, which resemble the challenges associated with the consolidation of the Brazilian Unified Health System (SUS, *Sistema Único de Saúde*) and the expansion of healthcare services [23].

Regardless of the type of BDS (I, II, or III), they are needed to work a minimum of 40 h per week, with the number of dental surgeons and assistants varying depending on the size of the service [24]. The decision regarding the weekly working hours of dental surgeons specializing in periodontology is the responsibility of the service manager, but it is expected that this choice aligns with the number of procedures necessary to achieve established goals. This is because the higher the professional’s workload in this specialty, the greater the number of patient appointments they can accommodate.

The lack of compliance with the working hours of dental surgeons in public dental services significantly hinders the production capacity and utilization of the service by users. This becomes the main organizational barrier that directly impacts the achievement of service goals [25].

It was observed that type III CEO showed superior performance in terms of the number of specialized procedures and were able to achieve the established goals in the field of periodontology. These results are in line with the research conducted by Da Silva and colleagues in 2021 [26], where it was observed that services with more dental chairs and a more skilled team recorded a higher volume of appointments.

The monitoring of goals was significantly associated with the tendency to achieve them, since the monitoring and planning of the provided services are of the utmost importance to achieve good results in the quality indicators and standards [27]. In terms of evaluation, this becomes relevant, considering that procedural issues inherent to the daily routine of the services can be used as support to guide the CEO team regarding the process of negotiating and contracting goals with managers as well as defining priorities for improving the service quality based on the recognition of the achieved results, whether they are effective or in need of improvement regarding the intervention strategies [27].

The evaluation instruments used, both the PMAQ-CEO and the AMAQ-CEO, indirectly contribute to achieving the goals and the number of specialized periodontal procedures. These assessments aid in formulating value judgments for the health units under investigation, supporting decision-making. Access and quality of CEOs, detailed based on the evaluated standards, pose challenging issues for management and need to be addressed. They demonstrate how actions and services are practiced in CEOs and how managers, workers, and users are involved [28].

As a limitation of this study, it is important to emphasize that the use of secondary data from information systems may be subject to recording errors, which can lead to underestimation or overestimation of study estimates. Furthermore, the use of performance indicators that rely solely on goal fulfillment, without considering changes in population demands that influence the demand for dental services, is also a point to be considered. On the other hand, among the study’s advantages, the evaluation of all existing CEO in Brazil stands out, as well as the use of the largest available database on procedures performed in specialized dental care, which currently represents the most comprehensive source of information on the subject in the country.

Future research should continue with the supervision and ongoing analysis of service indicators, prioritizing the investigation of the reasons behind their deficiencies. It is crucial to assess and propose measures to reverse this situation. Furthermore, it is imperative to evaluate the utilization of dental care, as the use of these services can highlight regional disparities in their availability and access.

## Conclusion

Although most CEOs do not perform all specialized periodontal procedures, more than half have achieved the targets set by the Ministry of Health. The provision of specialized periodontal services at the CEO is affected by the number of professionals and their training, as well as the availability of equipment. The presence of a prophylaxis device with a bicarbonate jet and a higher number of specialized professionals working at the CEO plays a crucial role in carrying out all specialized periodontal procedures. Regarding the achievement of goals, a positive influence is observed when the service is managed by someone with a background in public health or public management and when the weekly working hours of the periodontist align with the reality of the CEO.

## Data Availability

The datasets supporting the conclusions of this article are available in the second cycle of the External Evaluation of the National Program for the Improvement of Access and Quality in Specialized Dental Centers (PMAQ-CEO). The Brazilian Ministry of Health made the data available https://www.gov.br/saude/pt-br/composicao/saps/pmaq/ciclos-do-pmaq-ceo/2-ciclo-ceo/instrumentos-de-avaliacao-externa).
